# The role of metronomic capecitabine for treatment of recurrent hepatocellular carcinoma after liver transplantation

**DOI:** 10.1038/s41598-017-11810-z

**Published:** 2017-09-12

**Authors:** Matteo Ravaioli, Alessandro Cucchetti, Antonio Daniele Pinna, Vanessa De Pace, Flavia Neri, Maria Aurelia Barbera, Lorenzo Maroni, Giorgio Frega, Andrea Palloni, Stefania De Lorenzo, Maria Cristina Ripoli, Maria Abbondanza Pantaleo, Matteo Cescon, Massimo Del Gaudio, Giovanni Brandi

**Affiliations:** 1 Department of Medical and Surgical Sciences, S. Orsola-Malpighi Hospital, Alma Mater Studiorum, University of Bologna, V. Massarenti 9, 40138 Bologna, Italy; 2 Department of Experimental, Diagnostic and Specialty Medicine (DIMES), S. Orsola-Malpighi Hospital, Alma Mater Studiorum, Bologna University, V. Massarenti 9, 40138 Bologna, Italy; 3 “G. Prodi” Interdepartmental Center for Cancer Research (C.I.R.C.), University of Bologna, V. Massarenti 9, 40138 Bologna, Italy

## Abstract

The management of recurrent hepatocellular carcinoma untreatable with surgical options is based on systemic therapy with sorafenib. Due to the high rates of adverse events connected to the therapy with sorafenib, metronomic capecitabine seems a promising strategy for these patients. We analyzed the data of 38 patients with hepatocellular carcinoma recurrent after liver transplantation performed at our center. We compared the outcome of 17 patients receiving metronomic capecitabine versus 20 patients experiencing best supportive care and versus the data of the literature about treatment with sorafenib. In the group treated with metronomic capecitabine we observed an increased survival after tumor recurrence at the univariate and multivariate analysis compared to the group of best supportive care (median 22 months vs. 7 months, p < 0.01). Data from the literature on the use of sorafenib showed outcomes like our study group, with similar patient and tumoral features. The episodes of acute rejection and the tumor stage at the recurrence showed a correlation with patient survival at the univariate analysis. The metronomic capecitabine for hepatocellular cancer recurrent after liver transplantation seems effective without important adverse events and comparable results to sorafenib.

## Introduction

Hepatocellular carcinoma (HCC) is one of the main indications to liver transplantation (LT) in Western countries^1^. Tumor recurrence, which occurs in about 10–30% of recipients, remains one of the most important negative predictor of post-LT survival^2, 3^. The management of the recurrent HCC includes surgical resection and loco-regional treatments alone or combined with systemic therapies. In this perspective, sorafenib, an oral multiple-tyrosine kinase inhibitor, proved effective against advanced HCC in randomized clinical trials^4, 5^ and several small retrospective, heterogeneous studies reported a survival advantage in transplanted patients with HCC recurrence, when compared to best supportive care (BSC)^6–12^. However, its safety profile has raised some concerns in the setting of post-LT recurrence^13, 14^. The prevalent adverse events reported during sorafenib treatment were fatigue, dermatologic and gastrointestinal symptoms. In Sposito’s study, hand-food skin reaction was observed in 60% of patients; while diarrhea and fatigue were observed in 40% and 16.7%, respectively. All adverse events were grade 1–3 in severity^6^. A recent meta-analysis of 8 retrospective studies suggested a potential positive role of sorafenib in the post-LT setting, but the 1-year survival positively correlated with an increase in several adverse events: the median incidence for grade 3–4 dermatologic, gastrointestinal toxicity and fatigue were 22.5%, 18% and 16.1%, respectively^15^. All these figures were quite higher than those reported in randomized control trials (RCTs)^16^. The adoption of mammalian target of rapamycin (mTor) inhibitors may have a role in the treatment of HCC recurrence after LT, thanks to their dual effect of anti-angiogenesis and immunosuppression. Unfortunately, the real advantage of sirolimus has been proven in only one significant clinical series^17^.

Another treatment available in this setting is metronomic capecitabine (MC), which is the administration of a lower dosage of cytotoxic drugs in continuous without breaks^18^. In advanced HCC, MC showed a survival benefit in term of recurrence free survival (RFS) and overall survival (OS) in controlled large phase II study, both in first and second line^19^. Some recent studies confirm the survival benefit of MC in patients previously treated with sorafenib or intolerant to sorafenib^20, 21^ and the adverse events were lower than those reported with standard chemotherapy^22, 23^. This treatment has a low toxicity profile with few adverse events and no reported cases of dose reduction or treatment discontinuation due to side effects.

In the present study, we explored the putative efficacy of MC in HCC recurrence after LT. We compared the survival rates of patients treated with MC with a similar cohort who experienced BSC; we analyzed which of the several variables related to the tumor, the tumor treatment, the recipient and the immunosuppressive treatment were associated to the improved survival. Furthermore, the results of the present study group were compared to the series reported in the literature regarding the employment of Sorafenib.

## Results

**Figure 1:**
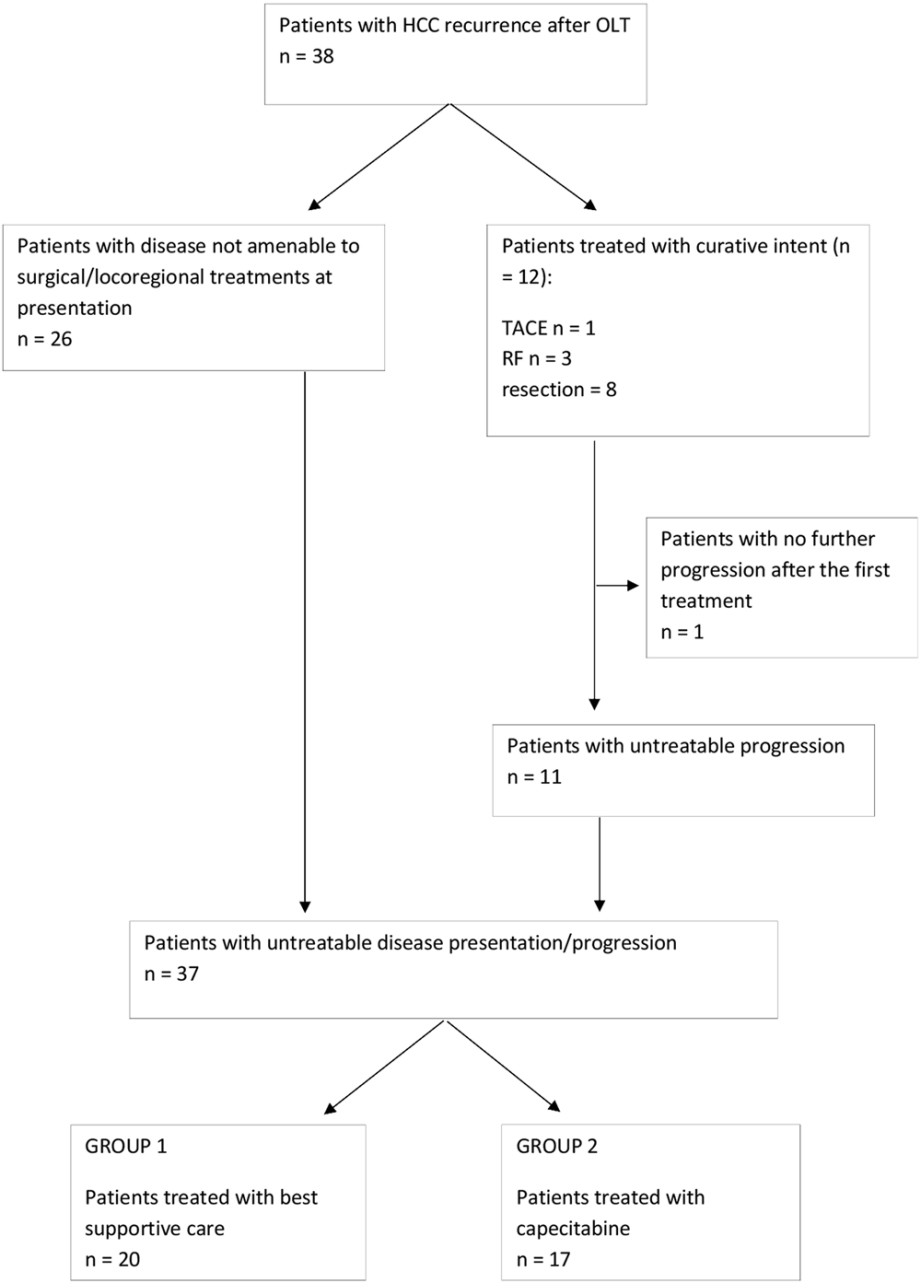
Flow chart descriptive of the patient distribution among patients.

From January 1997 to January 2012 a total of 48 patients experienced HCC recurrence after LT. Out of this pool, we considered 38 patients for whom the data collection was exhaustive. Eight of these patients had a recurrence treatable with surgical resection and 4 with radiofrequency (RF) or trans arterial chemoembolization (TACE). Of this group, 1 patient did not show any recurrence or disease progression after resection while all the others had an untreatable disease progression. The other 26 patients had an untreatable disease at the time of presentation. Finally, a total of 37 patients with untreatable disease then underwent either chemotherapy with Capecitabine (n = 17) or BSC (n = 20). The study design and patient grouping is depicted in Fig. 1.

### Groups’ characteristics

The baseline characteristics of the patients as well as the differences between the two groups are outlined in Table 1. Of note, that of all pre-LT and post-LT features collected, only the presence of micro-vascular invasion was significantly different in the two groups (43% study group vs. 85% control group, p = 0.013). The immunosuppressive treatment was not different between the two groups with respect to the introduction of mTOR inhibitors, however we observed an increased number of episodes of acute rejections on BSC group (33.3%) while no such events occurred in Capecitabine group (*p = 0.01*). Considering the adoption of other therapies, the rate of surgical resection of tumor recurrence was similar in patients before Capecitabine adoption and BSC adoption (15% vs. 29%, p = 0.4).

**Table 1:** Baseline characteristics of the patients in study.

	Total (37 pts)	BSC (20 pts)	Capecitabine (17 pts)	p value
Age at LT median (minimum-maximum)	52 (41–68)	52 (41–66)	53 (42–68)	n.s.
Gender (male)	32 (86.5%)	18 (85.7%)	14 (87.5%)	n.s.
MELD at LT median (minimum-maximum)	15.2 (7–39)	15.5 (7–39)	15.2 (7–27)	n.s.
CHILD at LT				n.s.
A	6 (16.2%)	3 (15%)	2 (11.8%)	
B	19 (51.4%)	12 (60%)	8 (47.1%)	
C	12 (32.4%)	5 (25%)	7 (41.1%)	
Etiology (HCV)	28 (75.5%)	15 (71.4%)	13 (81.3%)	n.s.
Treatment pre-LT	24 (75%)	16 (84.2%)	8 (61.5%)	n.s.
Pre-LT tumor stage at imaging within MC	22 (62.9%)	11 (55%)	11 (73.3%)	n.s.
Tumor stage at pathology after LT, within MC	7 (20.6%)	2 (10%)	5 (35.7%)	n.s.
Microvascular invasion after LT	23 (67.6%)	17 (85%)	6 (42.9%)	<0.05
Immunosuppression with mTOR inhibitors	18 (50%)	12 (60%)	6 (37.5%)	n.s.
Acute rejection	7 (19.4%)	7 (33.3%)	0 (0%)	<0.05
Time to recurrence (months)	13 (median)	13 (median)	14 (median)	n.s.
Tumor stage at recurrence Within MC	9 (25%)	2 (10%)	7 (43.8%)	<0.05
Pattern of liver recurrence, multiple localizations	27 (75%)	17 (85%)	10 (62.5%)	n.s.
Site of recurrence (extrahepatic)	22 (61.1%)	13 (65%)	9 (56.3%)	n.s.
AFP at recurrence (>400)	7 (25%)	4 (26.7%)	3 (23.1%)	n.s.
CHILD at recurrence				n.s.
A	30 (81.1%)	16 (80%)	15 (88.2%)	
B	5 (13.5%)	3 (15%)	2 (11.8%)	
C	2 (5.4%)	1 (5%)	0	
MELD at recurrence median (minimum-maximum)	10 (6–23)	10 (6–23)	9 (6–15)	n.s.
Liver Resection at the recurrence	8 (21.5%)	3 (15%)	5 (29.4%)	n.s.

The median time to recurrence was similar for all patients about 13–14 months, however the tumor stage at the recurrence was more often within Milan criteria in Capecitabine group: 43.8% vs. 10% in BSC group (*p* = *0.02*). The pattern (single lesion vs. multiple), site (intra vs. extrahepatic) of recurrence and level of alfafetoprotein (above vs. under 400) were instead not significantly different between the two groups.

### Survival analysis

**Figure 2:**
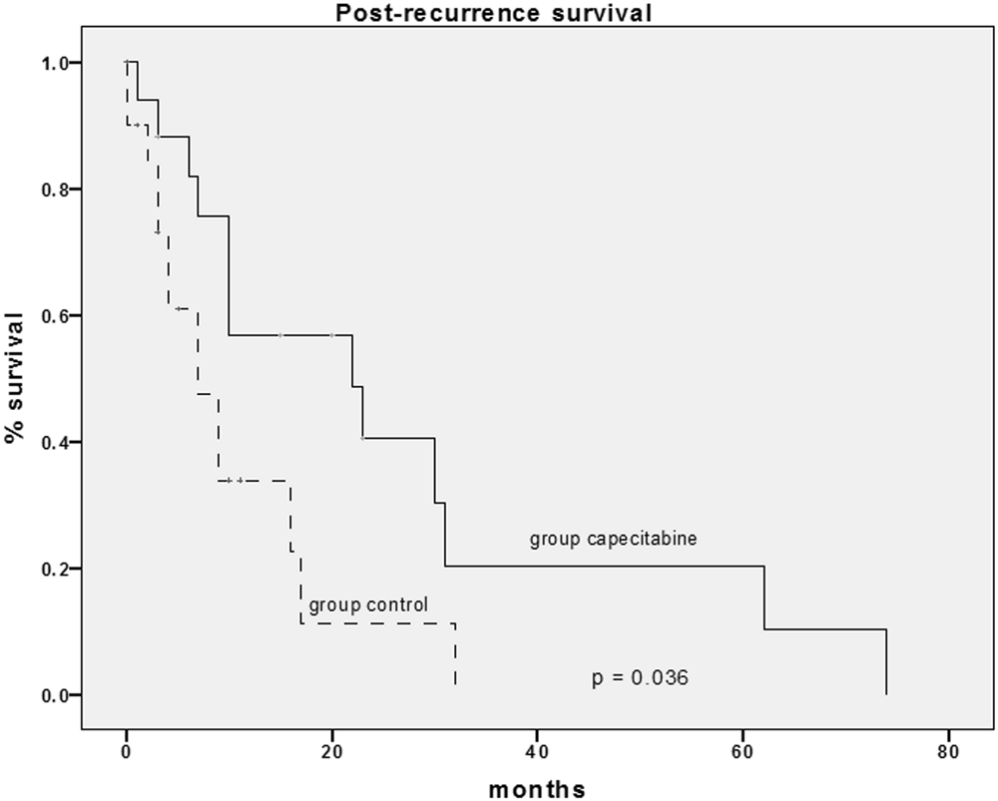
Analysis of the post-recurrence survival between group 1 (control) and group 2 (capecitabine).

The survival analysis conducted between the two groups showed an improved survival in the group treated with capecitabine in term of prolonged post-recurrence survival (Fig. 2). Patients of the BSC group had a median post-recurrence survival of 7 months versus 22 months in the Capecitabine group (*p = 0.036*). The univariate and multivariate analysis of the survival after HCC recurrence are reported in Table 2. While at the univariate analysis the absence of previous episodes of acute rejection, the recurrence within Milan criteria and the treatment with capecitabine were all significantly related to an increased survival, at the multivariate analysis the only independent variable associated to a better outcome was the treatment with capecitabine.

**Table 2:** Univariate and multivariate analysis of the correlation between post-recurrence survival and the analyzed independent variables.

Predictor	Category	Univariate	p value	Multivariate	p value
		Median survival (months)		Hazard Ratio (95% CI)	
Aetiology of disease	HCV	20	n.s.		
	other	16			
Gender	female	13	n.s.		
	male	17			
Pre-LT treatment	no	13	n.s.		
	yes	16			
Tumor stage (histology)	inMC	18	n.s.		
	outMC	17			
Vascular invasion	absent	21	n.s.		
	present	17			
mTor inhibitor	yes	19	n.s.		
	no	14			
***Episodes of acute rejection***	***yes***	***6***	<***0.01***		***n.s*** *.*
	***no***	***20***			
***Tumor stage at the recurrence***	***outMC***	***12***	<***0.05***		***n.s*** *.*
	***inMC***	***33***			
Time to recurrence	<12 months	12	n.s.		
	>12 months	21			
Site of recurrence	hepatic	24	n.s.		
	extrahepatic	14			
AFP at the recurrence	<400	20	n.s.		
	>400	11			
Primary treatment at recurrence	other	17	n.s.		
	resection	30			
***Treatment after untreatable progression***	***BSC***	***7***	<***0.005***	***2.88 (1.277***–***6.504)***	<***0.01***
	***Capecitabine***	***22***			

When Inverse-probability weights regression was applied, adjusting for tumor features at recurrence, the estimated average time to a death in patients receiving BSC was 8.4 months (95% C.I.: 5.4–11.5) whereas, if all patients in the population were submitted to capecitabine, the average time to death was estimated to be 15 months more than when all patients were submitted to BSC (95% C.I.: 4.7–25.4; p = 0.004).

The predominant side effect was grade 1–2 hand-foot skin reaction, fatigue, epigastric pain (in only 2 patients) and, less frequently, hematologic toxicity (in particular anemia). No grade 3–4 adverse events were reported in the group treated with capecitabine.

### Comparison with the series published treated with sorafenib

Literature search retrieved 8 articles investigating the adoption of sorafenib of HCC recurrence after LT for a total of 120 individual patients. A summary of main characteristics and results is reported in Table 3 and the pooled analysis is reported in Table 4. As can be noted, compared to the present series, patient age, the prevalence of extra-hepatic recurrence, the adoption of additional therapies and the introduction of mTOR inhibitors were not significantly different between sorafenib patients and the present series. These figures resulted in non-significant differences in term of 1-year overall survival between capecitabine and sorafenib patients.

**Table 3:** Comparison of the patient features among the study group (capecitabine treatment) and the series of the literature (sorafenib treatment).

Author	Year	N pts	Mean age ± SD	1-year survival rate	N pts 1-year survival	N pts Extra-hepatic recurrence	N pts additional_treatments (no CHT)	N pts with mTOR
Present capecitabine	2016	17	54,0 ± 1,5	68,5%	12	9	7	6
Tan	2010	10	46,3 ± 6,0	55,2%	6	1	10	—
Yoon	2010	13	49,0 ± 1,4	40,0%	5	11	9	1
Gomez-Martin	2012	31	53,6 ± 1,6	65,0%	20	6	0	30
Staufer	2012	13	58,0 ± 1,5	69,0%	9	12	8	9
Vitale	2012	10	59,0 ± 4,9	63,0%	6	5	5	7
Sposito	2013	15	50,5 ± 2,8	67,7%	10	9	12	7
Waghray	2013	17	57,8 ± 1,5	62,0%	11	10	9	10
Zavaglia	2013	11	57,0 ± 9,0	18,0%	2	8	5	7

**Table 4:** Pooled sorafenib treatment vs. capecitabine treatment.

Pooled Analysis	N pts	Sorafenib	N pts	Capecitabina	Effect size	p-value
Age	120	53,8 yrs (51,0–56,7)	17	53,5 yrs (49,3–57,7)	0,014	0,451
Extra-hepatic recurrence	120	56,2% (34,3–76,0)	17	52,9% (29,2–76,6)	0,043	>0,999
Additional treatments	120	57,6% (38,7–74,6)	17	41,1% (17,7–64,5)	0,367	0,297
Swith m-TOR	110	61,7% (40,4–79,2)	17	35,3% (12,6–58,0)	0,597	0,122
1-year survival	120	57,7% (46,5–68,2)	17	68,5% (46,4–90,6)	0,251	0,431

## Discussion

Surgical resection and ablative strategies offer the best survival results in post-LT HCC recurrence, but these treatments are not always feasible. Moreover, the further relapse after liver resection is usual: in these cases, other treatments are needed to improve the clinical conditions and possibly the survival. There is an urgent need to integrate systemic therapy in a management algorithm of HCC relapse post-LT. The present study offers an alternative strategy to sorafenib for the treatment of unresectable HCC recurrence after LT. The major experience in this field is based on published case-control series or case reports since there are no uniform guidelines that could drive the clinical practice. Sorafenib has been the mainly tested drug since the Barcelona Clinic Liver Cancer (BCLC) group has recommended its use for advanced HCC in the latest consensus conference. The authors suggested the potential employment of sorafenib also in post-LT setting, but with weak evidence^24^.

In the meta-analysis by Mancuso and colleagues, sorafenib appears to be roughly safe and effective in the post-LT setting: the pooled assessment of the 1-year survival rate was 63% (ranging from 18% to 90%). However, these findings should be taken cautiously because of the heterogeneity and low quality of the evaluated studies^15^. Other chemotherapy protocols have been employed with some success in term of prolonged survival and in particular of different profile of toxicity^25, 26^.

The metronomic treatment is based on the principle of administering low doses of chemotherapeutic drug continuatively for long periods of time. This method showed an increased antitumor activity *in vitro* and *in vivo* through the inhibition of neo-angiogenesis and at the same time a reduced toxicity due to the reduced dose of drug at each administration^27^. Capecitabine, orally administered in metronomic fashion, is generally well tolerated by compensated cirrhotic patients both in first and in second line^19^. Although capecitabine has not been approved for advanced HCC, a large phase II study showed good results in advanced HCC patients of the same type as those included in the trials and treated with sofarenib. In addition, the activity of the capecitabine is further demonstrated by durable complete responses. In this setting, the adverse events were fatigue (23.7%), hand-foot skin reaction (16.9%), epigastric pain (11.9%) and limb edema (11.9%). The majority of these side effects were grade 2 in severity^28^.

On the wave of the good results reported by our center in treating HCC in cirrhotic patients, we tested MC in the transplantation setting, on patients who had HCC recurrence after LT. When we started with our protocol there were only retrospective studies and there was not any guideline to treat such type of patients. Furthermore, there were few data concerning the treatment toxicity in immunosuppressed patients. Our population treated with metronomic capecitabine had an improved survival, in comparison to the population treated with the only BSC and no major adverse event occurred in this population.

We observed a different tumoral profile in the two groups of our study at the time of liver transplantation, but the pattern of HCC recurrence was similar in term of extra-hepatic disease, time to recurrence after LT and AFP level (Table 1). Furthermore, an inverse probability weights to adjust multivariate analysis was applied to eliminate this selection bias.

The study by Mazzaferro groups published in 2013 comparing BSC versus sorafenib in transplanted patients showed a survival benefit for the treated group since the median survival after untreatable progression in that group was 21 months versus 2.2 months in the control group. The median survival observed in our study was similar; after recurrence, it was 22 months for the treated group versus 7 in the control group^6^. Interestingly, the pathological and clinical features of our cohort showed a more aggressive tumor compared to the population on that study. In Mazzaferro’s study the tumor stage was within Milan criteria in 53.8% of cases versus 20% of ours; the microvascular invasion also was present in only 36% of their patients, which represents half our rate. In our series, the disease free survival after the LT was very short and consistent in both groups (13–14 months). The populations in Mazzaferro’s paper had significant differences in time to recurrence between the two groups, which was almost double in the treatment group (38 months)^19^. In accordance with this study, our results showed an improved survival in the study group after HCC recurrence at the multivariate analysis independently by the tumor stage (Table 2).

The further comparison to the other series reported in the literature did not show any different 1-year patient survival among our study group treated with capecitabine and the other patients treated with sorafenib. The analysis was performed considering the confounding variables such as the recipient and tumor features, as reported in Tables 3 and 4. This statistical analysis from the literature review was performed to balance the problem related to the limited number of cases in our mono centric study.

The toxicity observed in the present study was lower than that reported in our previous article studying the efficacy of metronomic capecitabine in advanced HCC treated with MC, perhaps because in the present study the patients are no longer cirrhotic.

Since the indications to LT for HCC are expanding we should expect if not an increase, at least the persistence in the actual rate of HCC recurrence after LT. The investigation of new types of systemic treatment effective in this difficult category of patients is strongly needed and possibly also the use of adjuvant treatment before any HCC recurrence.

A striking relevance obtained the incidence of episodes of acute rejection in our study. It appeared that such events had happened more frequently in the control group. Our explanation for this observation was that it possibly related to the high dose of steroids and immunosuppressive drugs administered acutely to treat an episode of rejection; this might have increased the severity and incidence of HCC recurrence in these patients, similarly to what happens for the hepatitis C virus (HCV) recurrence after LT^29^. The management of the immunosuppression becomes very important in this setting not much for the introduction of mTOR inhibitors, which have not shown correlation with the post-recurrence survival, but because we need to find a balance between the graft protection and an immune system reactive against potential tumor cells circulating systemically. In this perspective, the development of laboratory assays able to detect the metabolic activity of the immune cells might be a very useful innovation, since often the blood level of the immunosuppressive drug alone cannot reveal the real status of the immune system of the transplanted patient^30, 31^.

## Conclusion

In conclusion, the metronomic capecitabine treatment for HCC recurrence after liver transplantation was a safe treatment, it seemed to improve the recipient survival compared to the best supportive care and it obtained a similar survival to the group treated with sorafenib as previously described, even if these data need a confirmation by other studies.

The innovation of our proposal lays in the metronomic administration, which could preserve the efficacy of the compound and at the same time reduces the toxicity, allowing the continuation of the therapy and the increased rate of success.

With the limitations, due to the retrospective nature of our study and the limited population number, MC seems to be a good candidate also for the treatment of patients with post-LT recurrence, due to a survival benefit and an acceptable safety profile, offering a good basis on which new randomized prospective clinical trials should be undertaken to compare the efficacy and drawback of sorafenib versus metronomic capecitabine.

## Patients and Methods

We analyzed data from a prospectively collected database; patients with recurrent HCC after LT performed at our medical center from January 1997 to January 2012 were included. The considered variables were: demographics, etiology of the underlying liver disease, number and type of pre-transplant HCC treatment, model for end-stage liver disease (MELD) and CHILD-PUGH score at the time of transplantation, radiological staging at the time of transplantation, histological staging on the native liver, main immunosuppressive regimen, use of mTOR inhibitor, episodes of rejection, date of HCC recurrence, MELD, CHILD-PUGH score and value of alfafetoprotein (AFP) at the recurrence, pattern and site of the recurrence, number and type of first recurrence treatment.

The study protocol was approved by the local medical ethics committee of the Bologna University Sant’Orsola-Malpighi Hospital and informed consent was obtained by all patients.

At the time of the study period we decided to use the present protocol and no other treatments, such as sorafenib, because there were only retrospective studies and there was not any guideline to treat such type of patients. The data regarding the efficacy of any treatment and the toxicity related to the use of immunosuppressant were not consistent to suggest a specific protocol.

Data collection and clinical activity performed were conducted in accordance with the institutional guidelines.

The radiological and histological staging prior to transplantation was classified as either within or without Milan criteria^32^. AFP value was classified as inferior or equal/superior to 400 ng/mL. The cut-off of the AFP-level was established according to previous reports^6^. The pattern of recurrence was described as single or multiple lesions and the site as intra or extra-hepatic. We recorded the status of the patient as deceased or alive at the last time of follow up. The patients were considered as treatable or untreatable at the time of recurrence of the HCC based on the possibility of liver resection. The patients treated with liver resection were included in the analysis at the moment of HCC recurrence. All the considered patients were divided into two groups according to differences in treatment when HCC recurrence was deemed untreatable: the control arm where only BSC was adopted for the management of the patients and the treatment arm where metronomic capecitabine was administered.

Immunosuppression was based on calcineurine inhibitors and steroids tapered within the first post-transplant month; the main immunosuppressor was cyclosporine until 2003 and tacrolimus thereafter^33^. The immunosuppressive regimen was switched from calcineurin inhibitors to mTOR inhibitors after diagnosing the HCC recurrence and in these cases sirolimus was adopted (Rapamune; Pfizer, target through level of 4–10 ng/mL). In few cases the shift to mTOR inhibitors occurred before the HCC recurrence due to the presence of a high tumor grade at the histology on the native liver, and everolimus was administered to these patients (Certican, Novartis: target through level: 4–10 ng/mL).

Patients transplanted for HCC were monitored with semestral thoraco-abdominal computed tomography (CT) scans for the first 3 years and then annually alternating with contrast enhanced ultrasound (CEUS) and chest X-ray. Each case was discussed multidisciplinarily with the intent of a radical cure whenever possible through either resection or loco-regional treatment. The response to the treatment was assessed retrospectively with the modified response evaluation criteria in solid tumors (RECIST). After the 2004, many of these patients who presented with untreatable recurrence of disease or progression were managed with administration of capecitabine, on the basis of the good results from our previous experience in advanced HCC setting^19^. Capecitabine was administered at metronomic dosage (500 mg twice daily in continuous) according to our previous study. Capecitabine was discontinued only when disease progression, according to mRECIST criteria, occurred during the treatment or when drug-related toxicity was evident. Monthly clinical visits and laboratory analysis were performed during the treatment on an outpatient basis and an imaging study was performed every 3 months.

### Statistical analysis

The descriptive statistic of the baseline characteristics was expressed with median and interquartile ranges for continuous variables and with percentages for categorical ones. We used Pearson’s Chi square test for categorical variables and the median test for continuous variables, to compare the considered characteristics between the two groups of patients (treated with best supportive care versus capecitabine). The survival after untreatable disease recurrence/progression was calculated with the Kaplan–Meier method starting from the date of HCC recurrence to the date of death or to the most recent follow-up visit. The chi-square test and logistic regression were used to assess the accuracy of variables as predictors of patient survival after post-LT tumor recurrence. Differences were compared by the log-rank test and variables were evaluated in the multivariate analysis using Cox’s proportional hazard model. Differences were considered significant for p-values less than 0.05. Statistical analysis was performed with SPSS (SPSS Base 10.0; Application Guide, SPSS Inc., Chicago, IL, 1998).

The relative small sample size does not allow to handle for possible covariate confounding. We thus used inverse probability weighting (IPW) approach. In particular, we estimated the treatment effect of Capecitabine over BSC via IPW. IPW estimators use estimated probability weights to correct for the missing-data problem arising from the fact that each subject is observed in only one of the potential outcomes. The propensity score was generated including TNM, pattern of recurrence and presence of extra-hepatic metastases and IPW was calculated appropriately. Then the package “teffects ipw” package for STATA was applied to estimate the benefit obtainable from Capecitabine over BSC when handling for covariates.

### Comparison to the data from the literature

A literature review was performed through Pubmed database using the following terms; “hepatocellular carcinoma”, “liver transplantation”, “sorafenib”, “tumor recurrence” until September 2016. Briefly, after abstract selection and full reading of those manuscript considered for relevance, 8 studies, investigating the effect of sorafenib for HCC recurrence after liver transplantation, were selected for comparison to the present data from capecitabine therapy. The main outcome measure was the 1-year survival since recurrence diagnosis. From the selected articles, clinical and tumoral features available, as well as 1-year survival rates, were meta-analyzed using the Dersimonian and Laird random effects model^34^. When patient survival was reported as median value, an exponential decline was assumed and 1-year survival rate extracted using the DEALE method^35^. Continuous data presented as medians were transformed in means and standard deviations using the formula proposed by Hozo^36^. Finally, comparison between pooled analysis of sorafenib patients and present patient population receiving capecitabine was accomplished by Fisher exact test or Student t-test analyses and by estimating the effect size, a measure unaffected by the sample size. Effect size values were calculated according to the formulas proposed by Lipsey and Wilson^37^. As rule of thumb, effect size values < |0.1| indicate negligible differences; values between |0.1| and |0.3| indicate small differences, values between |0.3| and |0.5| indicate moderate differences and values > |0.5| indicate large differences^38^.

### Data avalability statement

The datasets generated and analysed during the current study are available from the corresponding author on reasonable request.
